# Psychedelics and health behaviour change

**DOI:** 10.1177/02698811211008554

**Published:** 2021-05-29

**Authors:** Pedro J Teixeira, Matthew W Johnson, Christopher Timmermann, Rosalind Watts, David Erritzoe, Hannah Douglass, Hannes Kettner, Robin L Carhart-Harris

**Affiliations:** 1 CIPER - Faculty of Human Kinetics, University of Lisbon, Cruz Quebrada, Portugal; 2 The Synthesis Institute B.V, Amsterdam, The Netherlands; 3 Center for Psychedelic and Consciousness Research, Johns Hopkins University, Baltimore, USA; 4 Imperial College London Psychedelic Research Group, London, UK

**Keywords:** Psilocybin, serotonin, therapy, public health, interventions

## Abstract

Healthful behaviours such as maintaining a balanced diet, being physically active and refraining from smoking have major impacts on the risk of developing cancer, diabetes, cardiovascular diseases and other serious conditions. The burden of the so-called ‘lifestyle diseases’—in personal suffering, premature mortality and public health costs—is considerable. Consequently, interventions designed to promote healthy behaviours are increasingly being studied, e.g., using psychobiological models of behavioural regulation and change. In this article, we explore the notion that psychedelic substances such as psilocybin could be used to assist in promoting positive lifestyle change conducive to good overall health. Psilocybin has a low toxicity, is non-addictive and has been shown to predict favourable changes in patients with depression, anxiety and other conditions marked by rigid behavioural patterns, including substance (mis)use. While it is still early days for modern psychedelic science, research is advancing fast and results are promising. Here we describe psychedelics’ proposed mechanisms of action and research findings pertinent to health behaviour change science, hoping to generate discussion and new research hypotheses linking the two areas. Therapeutic models including psychedelic experiences and common behaviour change methods (e.g., Cognitive Behaviour Therapy, Motivational Interviewing) are already being tested for addiction and eating disorders. We believe this research may soon be extended to help promote improved diet, exercise, nature exposure and also mindfulness or stress reduction practices, all of which can contribute to physical and psychological health and well-being.

## Introduction

Promoting healthy lifestyles in areas such as diet, physical activity, smoking and drinking has become a key priority area in the public and private sector, with the present wellness market reportedly valued at US$4.5T (McGroarty, 2018). Relevant to this industry is the science of ‘health behaviour change’ devoted to exploring and expanding the understanding of theories, determinants and interventions in this area (e.g., Davidson and Scholz, 2020). Promoting healthier lifestyles is important as unhealthy behaviours increase the probability of manifest disorders such as obesity, diabetes, depression, cancer and cardiovascular disease, as well as untold personal and familial suffering and significant economic burden (World Cancer Research Fund/American Institute for Cancer Research, 2018). Healthy lifestyles and mental health are closely related (e.g., Nagasu et al., 2019) and unhealthy lifestyle choices can cross over into pathology, with substance abuse being a major contributor to premature death (Case and Deaton, 2015). Tobacco and alcohol use in particular constitute a substantial health burden, together accounting for approximately 15% of global deaths (World Health Organization, 2017b, 2018). Both obesity (World Health Organization, 2020) and physical inactivity (Ding et al., 2016) remain highly prevalent worldwide, despite several decades of public health campaigns and policies intended to combat them.

Psychedelic^1^ compounds such as LSD (lysergic acid diethylamide), psilocybin (present in ‘magic mushrooms’) and DMT (dimethyltryptamine, present in ayahuasca^2^ and other preparations) may not seem like obvious tools for promoting healthier living. However, plants and fungi with psychedelic potential have been used by humans for centuries, if not millennia, for holistic ‘healing’ of body and mind (Akers et al., 2011; Lowy, 1971; Schultes, 1969). In the mid-20th century, aided by the laboratory synthesis of mescaline, LSD and psilocybin, scientists began to systematically test the effects of these compounds, with hundreds of studies performed. Several of these focused on the ability of psychedelics to promote traditional psychotherapy—with positive results claimed in the treatment of mood disorders and addictions, in particular. For example, a systematic review of 21 studies published between 1949 and 1973 covering the use of LSD and psilocybin for treating depression reported that 79% of participants had clinician-judged improvement after treatment (Rucker et al., 2016). In this period, over 40,000 patients participated in more than 1000 published studies with psychedelics, with many encouraging outcomes (Grinspoon, 1981; Grinspoon and Bakalar, 1979). Circa 1970, psychedelic research was halted due to prohibitive new legislation with formidable barriers to research, inaccessibility of drug product, a shifted public opinion, professional marginalization and lack of funding (Bonson, 2018; Johnson et al., 2008).

The 21st century has witnessed a so-called ‘renaissance’ of human research with psychedelic compounds. This is reflected in mainstream coverage of the topic, advocacy movements (e.g., Mazzei, 2019) and improved funding for clinical research (e.g., Emerson et al., 2014; Nichols, 2014; Sessa, 2018). Research of psychedelic-assisted psychotherapy has been published or is now being planned or underway for a range of psychiatric conditions including cancer-related distress (Gasser et al., 2014, 2015; Griffiths et al., 2016; Grob et al., 2011; Ross et al., 2016), depression (e.g., Carhart-Harris et al., 2021; National Library of Medicine [NLM], 2017a, 2019b), anxiety (NLM, 2017b), substance use disorders (e.g., Bogenschutz et al., 2015; Johnson et al., 2014; Moreno et al., 2006), obsessive compulsive disorder (Moreno et al., 2006) and eating disorders (Carhart-Harris et al., 2021). Despite mostly small-sample studies, published results are largely promising (Johnson and Griffiths, 2017). In the USA, the FDA has granted ‘Breakthrough Therapy Designation’ to psilocybin therapy for depression for two separate sponsors, effectively fast-tracking the development pathway to licensing if clear criteria are met. The substances ketamine and MDMA (3,4-methylenedioxymethamphetamine), which can induce psychedelic-like effects, are also being studied for treating depression, posttraumatic stress disorder and substance abuse (e.g., Krediet et al., 2020). MDMA has also been granted Breakthrough Therapy Designation by the US FDA for treating PTSD. However, because ketamine and MDMA are not considered ‘classic psychedelics’ (their mechanisms of action only partially overlap with those of psilocybin, LSD or DMT), they will not be covered in this review.

## How do psychedelics work? A summary of neurobiological findings

Classic psychedelics are direct agonists at the serotonin (5-hydroxytryptamine, or 5-HT) 2A receptor (5-HT2AR) subtype, and while all of the most popular psychedelics also have activity at other receptor subtypes, agonism at the 5-HT2AR appears to be key to their main action (Glennon et al., 1984; Preller et al., 2017; Vollenweider, 1998; Winter et al., 2004). Psychedelics have a favourable toxicity profile and therapeutic index (Van Amsterdam et al., 2015), and apparently negligible addiction potential (Rucker et al., 2018). The main hazards relate to the intensity of the altered state of consciousness they produce, particularly at higher doses, and the need for professional supervision under these conditions (Johnson et al., 2008).

The effect of stimulating the 5-HT2AR is to increase the postsynaptic gain of the host neuron, which is commensurate with it becoming more excitable to input. 5-HT2ARs are most densely expressed on cortical layer V pyramidal neurons, important information integration units (Jakab and Goldman-Rakic, 1998). This increase in postsynaptic gain translates into a spike-wave decoherence at the population level (Celada et al., 2008) and increase in the complexity, unpredictability or entropy of field potentials—an effect that correlates closely with the intensity of subjective effects in humans (Schartner et al., 2017). Higher, macroscopic level effects include the dysregulation of large-scale intrinsic networks (Carhart-Harris et al., 2016), increase in the repertoire of high-spatial frequency global brain states (Atasoy et al., 2018), and an increase in the communication between large-scale intrinsic networks (Carhart-Harris et al., 2016; Tagliazucchi et al., 2016). At the anatomical level, psychedelics have been found to promote cortical synaptogenesis, an important marker of neuroplasticity (Ly et al., 2018). The pertinent question, however, is: how do these effects relate to the putative therapeutic action of psychedelic therapy?

Recently a model was introduced to directly address this question (Carhart-Harris and Friston, 2019). It takes inspiration from the hierarchical predictive processing model of global brain function, which states that the brain strives to model its world in order to minimize surprising exchanges with it and thus, promote its mastery. Applied to psychedelics, the so-called ‘REBUS’ model, ‘relaxed beliefs under psychedelics’ states that psychedelics decrease the prediction-weighting of priors at different levels of the brain’s functional hierarchy—but particularly its highest hierarchical levels. In plainer language, this relates to dysregulated activity in relevant dimensions of brain function thought to encode high-level predictive models, such as the strength of oscillatory rhythms (such as the predominant alpha rhythm) and the integrity of intrinsic brain networks (such as the default-mode network). In more intuitive experiential terms, this effect relates to a felt relaxation of beliefs or assumptions e.g., about one’s self, relationships with others, or to the world more generally. In many psychiatric disorders, it is argued that habits of mind and behaviour, as well as beliefs, become ‘too precise’, meaning they are rigidly encoded and too influential. Examples include the negative cognitive bias in depression, specific cravings in addictions, specific fears in anxiety disorders, specific obsessions in obsessive compulsive disorder and specific bodily beliefs in body image disorders. According to the REBUS model, psychedelics afford the individual respite from weighty beliefs and thus a window of opportunity for change that can be exploited if combined with a commitment to therapeutic development (Carhart-Harris, 2019).

The evidence presented above highlights a potentially relevant link between the neuroscientific findings and behavioural change which may be facilitated by psychedelics. Revision of high-order mechanisms not only have an introspective consequence (i.e., in affective and cognitive domains) but may also result in exploratory behaviour which is consistent with new patterns and beliefs acquired during or after the acute experience. For example, a new appreciation for natural environments (Lyons and Carhart-Harris, 2018) could result in an increase in nature immersion behaviours (e.g., choosing parks or riverside trails to be more physically active) or a more sustainable shopping and dietary pattern. Such an increase in exploration of new behaviours would be consistent with an increase in the personality domain called ‘openness to experience,’ which has been shown to be increased by psilocybin (MacLean et al., 2011). From a mechanistic point of view, evidence from psychology and neuroscience experiments show that psychedelics are able to induce flexible patterns of thinking in different cognitive domains, which occur at a basic level of perception (Kometer et al., 2011; Timmermann et al., 2018), as well as on domains related to language and semantics (Family et al., 2016). Psychedelics may also enhance creative thinking (Kuypers et al., 2016) during acute states, depending on the context in which the experience takes place (Hartogsohn, 2016). It is thus reasonable to assume that the effects of psychedelics at multiple levels of the cognitive hierarchy may result in long-term changes in behaviour.

## Could psychedelic experiences increase self-determination?

Self-determination, or perceived autonomy, can be defined as the degree of self-endorsement of one’s actions at the highest order of reflection (Ryan and Deci, 2006). Self-determined motivation is consistently associated with improved psychological well-being and with engagement with—and persistence in—a variety of tasks and long-term behaviours (Ntoumanis et al., 2020). Consequently, the concept has been explored in studies in health behaviour change, in areas such as dental hygiene (Halvari et al., 2019), diet, exercise and obesity (Teixeira et al., 2012a), diabetes (Phillips and Guarnaccia, 2020) and tobacco cessation (Williams et al., 2016), with generally encouraging results (Leblanc et al., 2016; Sheeran et al., 2020; Teixeira et al., 2012b). We propose that it could present a fruitful psychological framework from which to understand how psychedelics could assist in lifestyle change, especially from a motivational viewpoint.

According to self-determination theory (SDT; Ryan and Deci, 2017), at the root of self-determined motivation and behaviour is the satisfaction of the three psychological needs of competence, autonomy and relatedness (see Table 1 from Teixeira et al., 2020). Much the same as the five human needs, as defined by Maslow, may be necessary for human survival and development (Maslow, 1943), SDT posits that competence, autonomy and relatedness are innate essential *psychological* needs for mental health and for psychological growth. It suggests that human beings are naturally inclined to ‘engage in interesting activities, to exercise capacities, to pursue connectedness in social groups, and to integrate intrapsychic and interpersonal experiences into a relative unity’ (Deci and Ryan, 2000) but this will occur only to the extent basic psychological needs are satisfied. SDT also makes reference to the concept of ‘true’ or ‘core’ self,^3^ one which researchers and practitioners involved with psychedelic therapy will easily relate to many participants’ personal accounts (e.g., Noorani et al., 2018; Watts et al., 2017).

**Table 1.** Conceptual definitions of the three psychological needs from self-determination theory.

Psychological need	Conceptual definition
Autonomy	The psychological need to experience self-direction and personal endorsement in the initiation and regulation of one’s behaviour. The hallmarks of autonomy need satisfaction are volitional action and wholehearted self-endorsement (i.e., personal ownership) of that action.
Competence	The psychological need to be effective in one’s interactions with the environment. It reflects the desire to extend one’s capacities and skills and, in doing so, to seek out optimal challenges, take them on, and exert effort and strategic thinking until personal growth is experienced.
Relatedness	The psychological need to establish close emotional bonds and attachments with other people. It reflects the desire to be emotionally connected to and interpersonally involved in warm relationships. The hallmarks of relatedness need satisfaction are feeling socially connected and being actively engaged in both the giving and receiving of care and benevolence to the significant people in one’s life.

Conceptual definitions are based on Ryan and Deci (2017).

We hypothesize that competence (akin to a sense of self-efficacy and confidence in one’s capacities), autonomy (i.e., wholehearted self-endorsement of one’s actions) and interpersonal relatedness are all factors which psychedelics could plausibly influence. Although at present we have no direct evidence for such effects, accounts of participants in research trials for depression (Watts et al., 2017), alcohol cessation (Nielson et al., 2018) and smoking cessation (Noorani et al., 2018) suggest this could be the case. Regarding competence, Nielson et al. (2018) included a category of ‘confidence, motivation and resolve’ and another of ‘commitment to change’ as descriptive of participants’ experiences. In turn, Watts et al. (2017) reported feeling more ‘confident’, ‘resilient’ and ‘effective’. In relation to ‘autonomy,’ Noorani et al. (2018) found insights into self-identity (e.g., unveiling of true self, and honesty with oneself) to result from sessions, and Watts et al. (2017) reported increased connection to self and feeling more attuned with ones’ internal needs and inherent worth as central processes underlying transformative experiences, in previously depressed patients (Watts et al., 2017). For example, ‘One of the things that happened to me (…) was the magnification of how important it was to be true to yourself and have integrity about it’ (Noorani et al., 2018: 11).

Finally, regarding relatedness, Noorani et al. (2018) noted that sessions left participants with lasting impressions of interconnectedness and an increase in prosocial behaviour. ‘Relatedness’, as defined by SDT, is an especially interesting case vis à vis the evidence that significant psychedelic experiences are often associated with increased feelings of connectedness to others (Carhart-Harris et al., 2018; Watts et al., 2017). A sense of unity, which might be conceptualized as an ultimate level of relatedness, is one of the features of the mystical experience construct that is strongly affected by classic psychedelics (Johnson et al., 2019). Future research will be needed to investigate whether psychedelic experiences relate to changes in concepts central to SDT (e.g., need satisfaction and autonomous self-regulation), and to what extent these concepts may predict lasting behaviour change after a positive experience or therapeutic process.

## Spontaneous health behaviour changes with psychedelics

*As a result of these lessons [from psychedelic treatment for depression], there were some major lifestyle changes; nearly half of the sample reported improvements to diet, exercise, and cutting down on drinking alcohol. One described the improvement to his diet that happened after the dose as ‘life-changing’, although he was not sure how these changes came about as he did not receive direct ‘lessons’ about diet*. (Watts et al., 2017: 13)

To our knowledge, no study has been conducted specifically to investigate lifestyle behaviours such as over-eating and physical activity in relation to psychedelic use. However, some studies have asked participants to report spontaneous changes in various areas of their lives, including in health behaviours, perhaps due to the fact that anecdotal accounts of these phenomena are not uncommon. In fact, it appears that the first studies in alcohol abuse were initiated precisely because of *ad hoc* observations by some users – notably Leo Zeff, a famous psychedelic clinician, regarding his own smoking cessation success after using a classic psychedelic (c.f. Johnson et al., 2017b). It is also interesting to note that, along with environmental concerns, healthy eating and living more healthfully were popular elements of psychedelic culture from the beginning, at least in some communities (Smith and Sternfield, 1970).

In a recent observational study, 380 ayahuasca users were interviewed face to face and asked about their health (including height and weight, allowing for calculation of their body mass index (BMI)) and also their physical activity, diet and yoga/meditation habits (Ona et al., 2019). These measures were then compared to data from the general population. Although limited by the cross-sectional nature of the data (e.g., confounding factors may be present), results showed that ayahuasca users had a mean BMI of 22.6 kg/m^2^, well below the 30 kg/m^2^ cut-off for obesity and clearly lower than that of the general Spanish population (around 26 kg/m^2^ in 2016; World Health Organization, 2017a) and had a high fruit and vegetable consumption (60–75% ingesting 3–6 servings a day of each vs. 22–48% in the general population). As to physical activity, 55% reported being ‘as physically active as they wished’, a value that seems high but which cannot be compared with any standard measure of activity in the population (Ona et al., 2019).

In a US survey of 343 people who claimed to have stopped or reduced alcohol consumption and misuse after a psychedelic experience, 63% of the participants also endorsed ‘improved diet,’ and 55% reported ‘increased exercise’ as a result of their psychedelic experience (Garcia-Romeu et al., 2019b). In a similar study of 444 participants who claimed to have stopped or reduced cannabis, opioid or stimulant misuse after a psychedelic experience, 59% endorsed ‘improved diet’ and 58% endorsed ‘increased exercise’ as a result of their psychedelic experience (Garcia-Romeu et al., 2019a). It is unknown from these studies whether these individuals were intentionally seeking changes in these specific areas as they embarked on psychedelic use. However, one can speculate that many were probably not because only 9.9% reported an intention to reduce/quit drinking alcohol. This suggests that psychedelic-assisted interventions that more specifically target health behaviour changes could result in even higher rates of success in this domain.

The Johns Hopkins’ studies on smoking cessation also provide some evidence that participants were making other positive changes in their lives (besides reducing smoking) as they went through the psilocybin-assisted therapy programme. Both the initial (Johnson et al., 2014) and follow-up (Johnson et al., 2017a) studies report significant (51%) increases in a self-reported scale titled ‘positive behaviour changes’ as people moved from baseline to the end of the treatment. These were described further in a qualitative analysis, which reported increases in time spent in nature, taking time for oneself, prosocial behaviours such as volunteering and joining community groups, and greater engagement with art (Noorani et al., 2018). A similar pattern was observed in the study of psilocybin for depression in cancer patients, from the same research group (Griffiths et al., 2016). Although these are not health-related behaviours per se, results suggest psychedelics may be associated with life changes consistent with improved well-being and meaning. In support of this, a recent 4.5 year follow-up of cancer patients who underwent psilocybin-assisted therapy showed persisting increases in not just well-being/life satisfaction (reported by 86% of participants, *n*=12) but also in ‘positive behaviour changes’ attributed to the psilocybin experience (100% of participants, *n*=14) (Agin-Liebes et al., 2020).

A qualitative descriptive study with 16 participants who had been previously diagnosed with an eating disorder (ED, anorexia nervosa or bulimia nervosa) and were at various phases of illness and/or recovery investigated how having partaken in one or several ayahuasca ceremonies influenced their management of their condition or recovery process (Lafrance et al., 2017). A majority of participants reported reductions in ED-related negative thoughts, improvements in emotional processing and regulation, and an increased ability to identify what they perceived as the root psychological causes of the disorder. No specific behavioural changes were measured, but participants generally either reported more easily managing symptoms or having achieved full and sustained remission, subsequent to their psychedelic experience.

Finally, a recent study looked at benefits and challenges in 278 users of *microdosing* with LSD and/or psilocybin (Anderson et al., 2019). Microdosing typically involves the regular (i.e., 2–3 times a week) intake of about 5–15% of the normal dose used in most studies. Consequently, both the brain/neurological effects as well as the psychological (and phenomenological) effects with this dosing model are presumed to be partially different than those from taking a high dose (Passie, 2019). These differences notwithstanding, and also considering other limitations (self-selected convenience sample with no comparison group), microdosing was associated with spontaneous improvements in meditative practice (49.1% of participants), exercise (49.1%), eating habits (36.0%) and sleep (28.8%); and with reduced use of caffeine (44.2%), alcohol (42.3%) and tobacco (21.0%).

## From addiction to behaviour change interventions using psychedelics

Among the mental health/psychiatric targets of early clinical psychedelic research in the 1950s and 60s were disorders primarily defined by behavioural problems, including substance abuse disorders (e.g., alcohol, opioids). Surveying studies from the 1950s to 1970s, a meta-analysis of six double-blind intervention studies using LSD for alcohol misuse suggested a significant effect in abstinence rates at the short (2–3 months) and the medium-term (6 months), but not at 12 months (Krebs and Johansen, 2013). This line of research was resumed more recently, and results have tended to confirm the earlier observations with psilocybin for alcohol dependence (Bogenschutz et al., 2015), and extended it to tobacco smoking (Johnson et al., 2014, 2017b). In the latter studies, after two or three drug sessions and behaviour therapy before and after, biologically verified smoking abstinence at 12 months and ~30 months by far surpassed success rates in current treatment modalities (Johnson et al., 2017a). A complementary survey describes instances in which classic psychedelic use outside of research settings is followed by cessation or reduction of tobacco smoking (Johnson et al., 2017b).

Mechanisms of action in tobacco cessation with psychedelics reportedly include improved mood and affect, change in life priorities and values, motivational insights and emotional regulation (Johnson et al., 2017b). In addition, qualitative analysis of smoking cessation participants suggested psilocybin sessions helped in smoking abstinence by providing insights into self-identify and personal reasons for smoking, a sense of awe, curiosity and interconnectedness, and a persistent overshadowing of withdrawal symptoms (Noorani et al., 2018). It is noteworthy that for both smoking and alcohol dependence the most recent intervention trials using psychedelics have employed mainstream motivational/behaviour change methods such as Motivational Interviewing (Nielson et al., 2018) and Cognitive Behavioural Therapy (Johnson et al., 2014) to complement the substance-induced psychedelic experience(s). Both Motivational Interviewing and Cognitive Behavioural Therapy have been used extensively in the areas of mental health and health behaviour change (e.g., Frost et al., 2018; Health Quality Ontario, 2019).

The models of psychedelic therapy which are now emerging in the field (e.g., Watts and Luoma, 2020; Wolff et al., 2020) typically include one or two sessions where participants ingest the psychedelic compound (e.g., a psilocybin capsule), with variable amounts of preparation and integration therapeutic sessions (Johnson et al., 2008). Trained guides/therapists are usually present for the duration of the psychedelic experience. One reference is the use of psilocybin for smoking cessation at Johns Hopkins (Johnson et al., 2014, 2017a), where participants underwent a 15-week programme with six preparation/integration individual meetings. Psychedelic-assisted behavioural change interventions could also take place in a group format, which is often used in ceremonial settings, and holds much promise as a cost-effective way to deliver psilocybin therapy in research contexts. Although current lifestyle behaviour change interventions (e.g., for weight loss or diabetes) involve a wide variability of formats and length, a programme of several weeks (sometimes months) with regular educational sessions in a group setting is a very common model (e.g., Teixeira et al., 2010).

Looking forward, the therapeutic container for such work could be the ACE model (Accept, Connect, Embody) which was used to support patients undergoing psilocybin treatment for depression (Watts and Luoma, 2020) and is based on Acceptance and Commitment Therapy or ACT (A-Tjak et al., 2015). In fact, Walsh and Thiessen (2018) have also proposed that psychedelic therapy should incorporate Acceptance and Commitment Therapy, a framework which has also been used for different mental and physical health problems, including obesity (Lawlor et al., 2020) and depression (Bai et al., 2020). Alternatively, as highlighted above, Cognitive Behavioural Therapy (see also Wolff et al., 2020) and Motivational Enhancement Therapy (including Motivational Interviewing) could be used as therapeutic containers, as each of these has been supported by robust research as effective in supporting multiple varieties of health behaviour change (Miller and Rollnick, 2012). Motivational Interviewing in particular is largely consistent with SDT (Patrick and Williams, 2012), and both have a range of intervention techniques (e.g., Teixeira et al., 2020) which could be tested as part of future psychedelic-assisted behaviour change interventions.

## Final considerations

We have reviewed the modern resurgence in psychedelic research with a focus on therapeutic applications. Among these are the treatment of addictions (alcohol and tobacco) using psychedelics, which can be viewed as instances of health behaviour change facilitation. We have also briefly reviewed how biological effects of psychedelics may result in acute psychological effects, such as relaxed beliefs, that may have long-lasting effects on perspective and behaviour. In addition, we have proposed that SDT, which emphasizes autonomy, competence and relatedness, may be a useful framework to understand increases in internally driven motivation upon well-integrated psychedelic experiences. Finally, we propose directions for how psychedelic work in behaviour change may be conducted in the future, establishing several parallels between commonly used models, such as Cognitive Behavioural Therapy or Acceptance and Commitment Therapy, and emerging psychedelic-assisted therapies for tobacco cessation or depression (e.g., the ACE model). Another area for future enquiry is to examine the role of individual differences in suggestibility as well as drug-induced enhancements of suggestibility in moderating and mediating behavioural change (Carhart-Harris et al., 2015; Carhart-Harris and Friston, 2019).

One concern might be an impression by some in the public that administering psychedelics to patients might cause them to lose motivation for prior pursuits and for engagement in mainstream society, including abandonment of family responsibilities. We suspect such fears are driven not by the pharmacological effects of psychedelic drugs, but by their historical cultural association with the counterculture and their status as illicit drugs. The data that directly addresses these questions in clinical research do not show evidence for it. For example, Griffiths et al. (2006, 2011) examined ratings by community observers (i.e., family members, friends or co-workers with frequent contact with the participant) about the participant’s behaviours, attitudes and functioning. These were assessed before psilocybin administration and up to 2 or 14 months after psilocybin administration. Both studies showed significant long-term improvements as assessed by these community observers, suggesting that the intervention did not cause a general tendency for people to drop out of mainstream society or disengage with their families. The clinical studies reviewed have utilized preparation, monitoring and some form of integration (Johnson et al., 2008). As psychedelics move forward as possible approved therapeutics, to minimize the potential for societally disruptive effects it is important that clinicians observe appropriate clinical practice boundaries and not introduce counter-cultural or supernatural frameworks to patients in the context of psychedelic therapy (Johnson, 2020).

There are numerous future directions suggested by our framework. Studies should expand existing research to look at broader ranges of behavioural targets including diet, exercise and other ‘wellness behaviours’. If such research endeavours are also encouraging, this would further reinforce the already suggestive case that psychedelic therapy can be broadly applicable to behaviour change via transdiagnostic mechanisms. Future work should continue to conduct hypothesis-driven tests of the REBUS and other models that may be useful for bridging our understanding of how acute pharmacological effects translate into long-term behaviour changes. Empirical work should also address the role of therapy, for example, whether results are enhanced when psychedelic sessions are accompanied by explicit frameworks such as Cognitive Behavioural Therapy, Motivational Interviewing and the ACE model, versus psychedelic sessions with only general preparation, monitoring and follow-up discussion. Additionally, research should eventually compare these, and potentially other therapeutic models, in their ability to support psychedelic therapy for particular indications. From the broadest perspective, if psychedelic-assisted therapy is found to work through general processes, such as relaxed beliefs, psychological flexibility and self-determined motivation, this might be used to enhance behaviour change across a number of disorders and lifestyle challenges, and enhance the effects of multiple psychotherapeutic approaches. This would constitute a shift to a fuller transdiagnostic understanding of psychiatric disorders along a continuum with normative human behaviour and lifestyle challenges.

## Notes

1. The word ‘psychedelic’ is derived from the Greek meaning ‘mind manifesting’ or ‘mind revealing’.

2. A drink traditionally produced and used ceremonially in South America, but also used by churches in North America and Europe. It is commonly made from a combination of the *Banisteriopsis caapi* vine with one of several DMT-containing plants (such as *Psychotria viridis*) (Ott, 1996).

3. See Ryan and Deci (2017) for an in-depth discussion of this concept and its historical use in Psychology and Philosophy. In brief, it is conceived within the ‘self-as-process’ tradition as an innate, integrative, organizing ‘energy’ (motivational force) directed towards the realization of one’s potentials and full functioning, or eudaimonia. It is associated with the expression of integrity, spontaneity and vitality, and with a sense of ‘feeling real’, unmasked, ‘true to heart’, and with access and sensitivity to one’s feelings and needs.
